# Suppression of endothelial cell migration by tumor associated macrophage-derived exosomes is reversed by epithelial ovarian cancer exosomal lncRNA

**DOI:** 10.1186/s12935-017-0430-x

**Published:** 2017-06-06

**Authors:** Quanfeng Wu, Xiaoli Wu, Xiang Ying, Qinyi Zhu, Xinjing Wang, Lu Jiang, Xin Chen, Yueqian Wu, Xipeng Wang

**Affiliations:** Department of gynaecology and obstetrics, Xinhua Hospital, Shanghai Jiao Tong University School of Medicine, Shanghai, China

**Keywords:** Epithelial ovarian cancer, Tumor associated macrophage, Exosome, Endothelial cell, Migration

## Abstract

**Objective:**

To study the mechanism by which epithelial ovarian cancer (EOC)-derived exosomes restore the migration of endothelial cells that is suppressed by TAM-derived exosomes.

**Methods:**

Exosomes were isolated from TAMs in the ascites of patients with EOC. The effect of exosomes on the expression of endothelial cell miRNA was monitored by PCR. The miRNA mimics were transfected to explore their effects. Microarray data and literature searches were used to predict target genes and the impact of target gene pathways, and small interfering RNA was used to target these genes. We used migration assays to determine whether ovarian cancer cell-derived exosomes participate in the regulation of TAMs and endothelial cells. We used microarray data to identify the target lncRNA, and we constructed target lncRNA expression plasmids to validate targets by Western blotting.

**Results:**

We separated TAMs from the ascites of patients with EOC and isolated exosomes from TAM supernatants. After co-culture with HUVECs, these exosomes were efficiently incorporated into HUVECs. The migration of HUVECs was suppressed significantly in the exosome group compared with blank controls (P < 0.05).The miRNA mimic transfection and target gene prediction found that TAM-derived exosomes targeted the miR-146b-5p/TRAF6/NF-κB/MMP2 pathway to suppress endothelial cell migration; this result was supported by PCR and Western blotting analyses. The expression of exosomal miR-146b-5p isolated from serum in the EOC group was significantly increased compared to healthy individuals. Finally, TAM-derived exosomes and EOC SKOV3-derived exosomes in combination stimulated HUVEC cells and overcame the inhibition of endothelial cell migration caused by TAM-derived exosomes. Two lncRNAs that were carried by SKOV3-derived exosomes were identified as NF-κB pathway-associated genes by Western blotting.

**Conclusion:**

TAM-derived exosomes can inhibit the migration of endothelial cells by targeting the miR-146b-5p/TRAF6/NF-kB/MMP2 pathway. However, EOC-derived exosomes can transfer lncRNAs to remotely reverse this effect of TAMs on endothelial cells.

**Electronic supplementary material:**

The online version of this article (doi:10.1186/s12935-017-0430-x) contains supplementary material, which is available to authorized users.

## Background

Epithelial ovarian cancer (EOC) is considered the most malignant gynecological tumor; its mortality rate is the highest of all gynecological malignancies. The overall 5-year survival rate is approximately 30% [1]. A remarkable feature of advanced EOC is the presence of widespread peritoneal metastases at the time of initial diagnosis. However, the mechanisms of peritoneal seeding, spreading, and progression remain elusive.

Our previous studies suggested that various stromal cells, including activated endothelial cells, tumor-associated macrophages (TAMs), fibroblasts, and bone marrow-derived cells infiltrated the EOC peritoneum [2, 3]. More than 75% of the mononuclear immune cells in the peritoneum near a tumor implant were TAMs, which mimic chronic inflammation [2] and associated with tumor progression.

In a previous study, we demonstrated that CD68+ macrophages are in close contact with CD31+ endothelial cells in the peritoneum in the presence of EOC. We found that 53% of CD68+ cells and CD31+ endothelial cells display high levels of VCAM1 adhesion molecule expression, in contrast to 3.6% of CD3+ T cells [2]. Furthermore, it was suggested that the interaction of ovarian cancer cells and tumor-associated macrophages enhances the ability of endothelial cells to promote the progression of ovarian cancer [3]. However, how EOC cells regulate the interaction between TAMs and endothelial cells in the tumor microenvironment remains unknown.

Most recently, exosomes derived from multiple cells have been shown to play important roles in mediating communication between cells. Exosomes are released into the extracellular matrix after the fusion of multivesicular endosomes with the cell membrane, and have a diameter of approximately 30–100 nm. The exosomes carry microRNA (miRNA), long non-coding RNA (lncRNA), and other biologically active substances. One of our study showed that exosomes derived from EOCs could regulate the polarization of tumor-associated macrophages by transferring miR-223p [4].

In this work, the in vitro co-culture of TAM-derived exosomes with endothelial cells suppressed the migration of the endothelial cells. Thus, it seems that TAMs would not promote endothelial migration to participate in angiogenesis in the tumor microenvironment. However, when EOC-derived exosomes were added into the co-culture system, the migration of endothelial cells was restored, which indicated that EOC-derived exosomes play a central role in regulating the interaction of TAMs and endothelial cells.

Here, we investigate the mechanism and possible pathway by which EOC-derived exosomes restore the migration of endothelial cells that is inhibited by TAM-derived exosomes (Additional file 1).

## Results

### Identification of tumor-associated macrophages separated from epithelial ovarian cancer

**Fig. 1:**
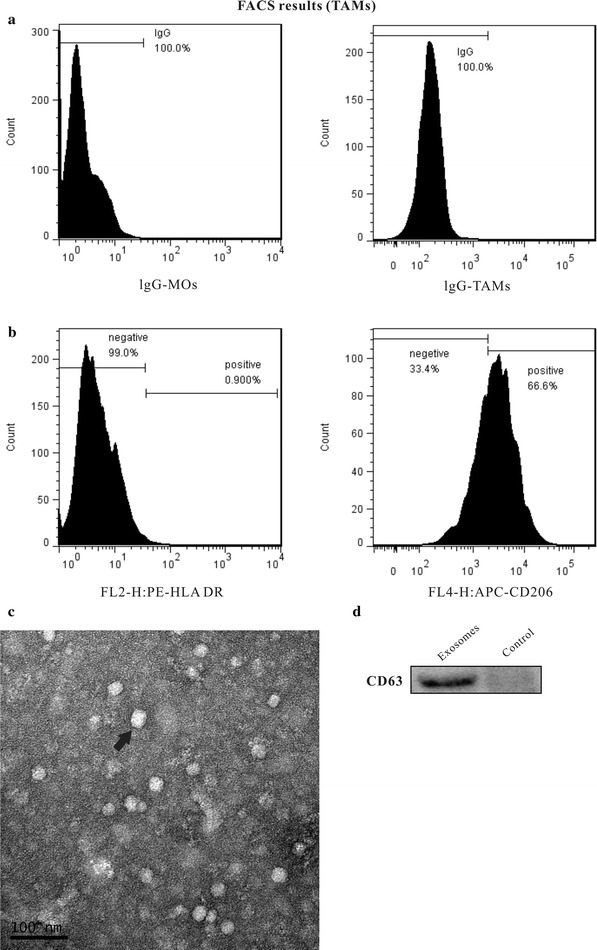
Identification of TAM-secreted exosomes. **a**, **b** TAMs were isolated from the ascites of EOC patients, and the percentages of HLA-DR and CD206-positive cells were determined by FACS. **c** Photomicrographs of exosomes derived from tumor-associated macrophages (TAMs) separated from epithelial ovarian cancer (EOC) fractionated by Exoquick. **d** Exosomal marker proteins in isolated exosomes were quantified by immunoblotting. Surface levels of the exosomal marker CD63 on the purified exosomes were measured

CD206 was used as a specific marker to identify TAMs. HLA-DR protein expression is decreased in TAMs, but it is expressed at much higher levels in CD14+ monocytes. We isolated the cells from the ascites of EOC patients with CD14+ magnetic beads. To identify whether these cells were TAMs, CD206 and HLA-DR protein expression was detected by flow cytometry. The portion of CD206+ cells was significantly higher (approximately 66.4% vs. 3.43%), and the rate of HLA-DR+ cells was significantly lower (approximately 0.900% vs. 86.2%) in TAMs isolated from the ascites of EOC patients compared with CD14+ macrophages (Fig. 1a, b).

Exosomes are characterized by their conserved size and density and the presence of specific protein markers [5, 6]. To ensure that exosomes were recovered and intact, Cryo-TEM images were used to confirm the secreted exosomes from TAMs. As previously reported [7–9], both samples contained small (30-nm diameter) and large (80-nm diameter) spherical exosomes (Fig. 1c). We then examined the expression of exosomal marker proteins in exosomes by Western blotting (Fig. 1d).

### Exosomes derived from tumor-associated macrophages are ingested into HUVECs

**Fig. 2:**
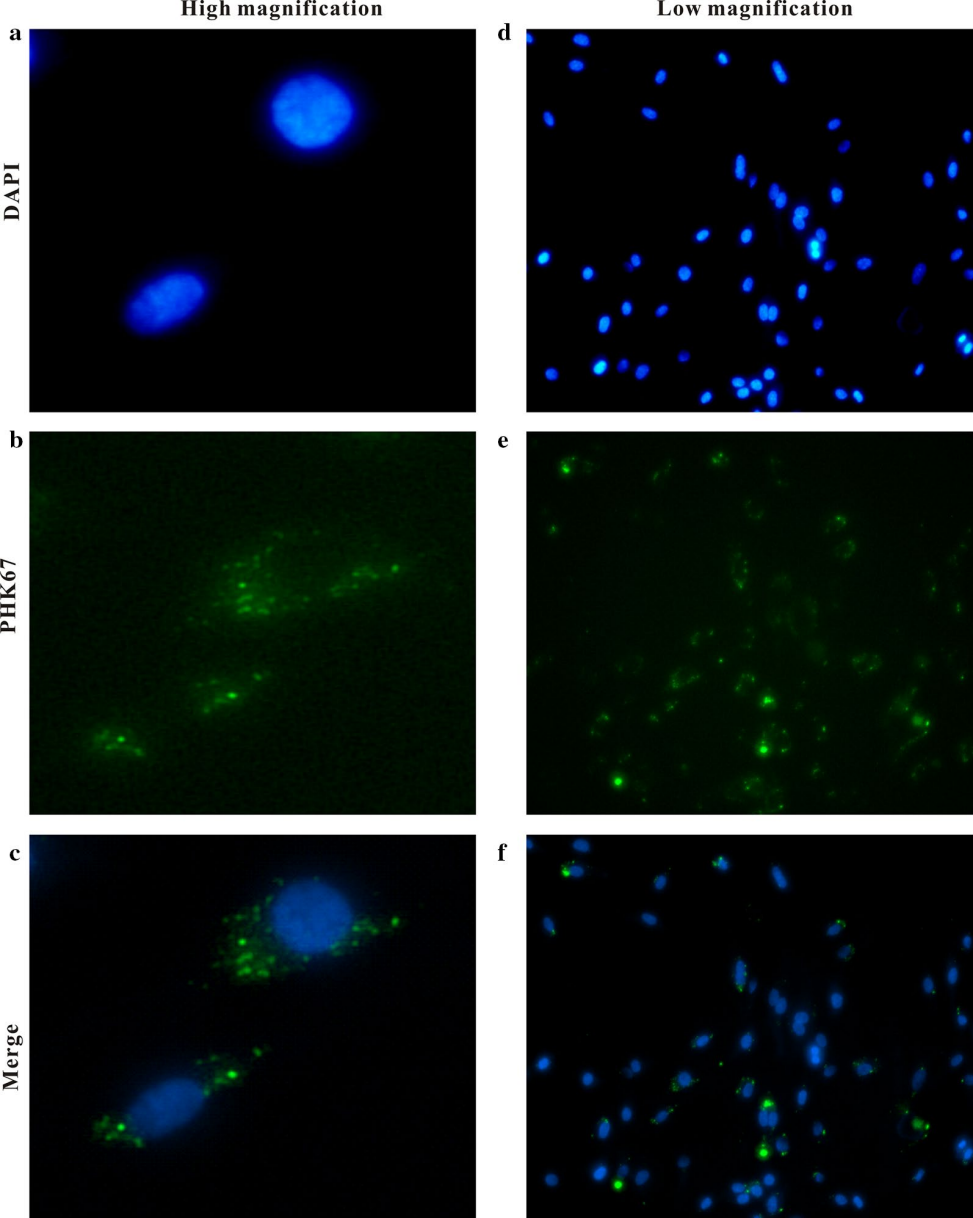
Internalization of the exosomes into recipient cells. HUVECs in culture were incubated with TAM-derived exosomes labeled with PKH67 (*green*). HUVECs were fixed with cold methanol and mounted with DAPI. High magnification images of HUVECs incubated with exosomes (**a**–**c**) or low magnification images of HUVECs incubated with exosomes (**d**–**f**)

To examine the potential internalization of exosomes by other cells, we labeled TAM-derived exosomes with the fluorescent dye PKH67. PKH67-labeled exosomes were incubated with HUVECs for 24 h, and the localization of exosomes was examined by fluorescent microscopy (Fig. 2). The internalization of PKH67-labeled exosomes (green) was observed as endosome-like vesicles in the cytoplasm of HUVEC cells. These studies indicate that TAM-derived exosomes can be ingested by other cells.

### miRNA expression in HUVECs in the presence or absence of TAM-derived exosomes

**Fig. 3:**
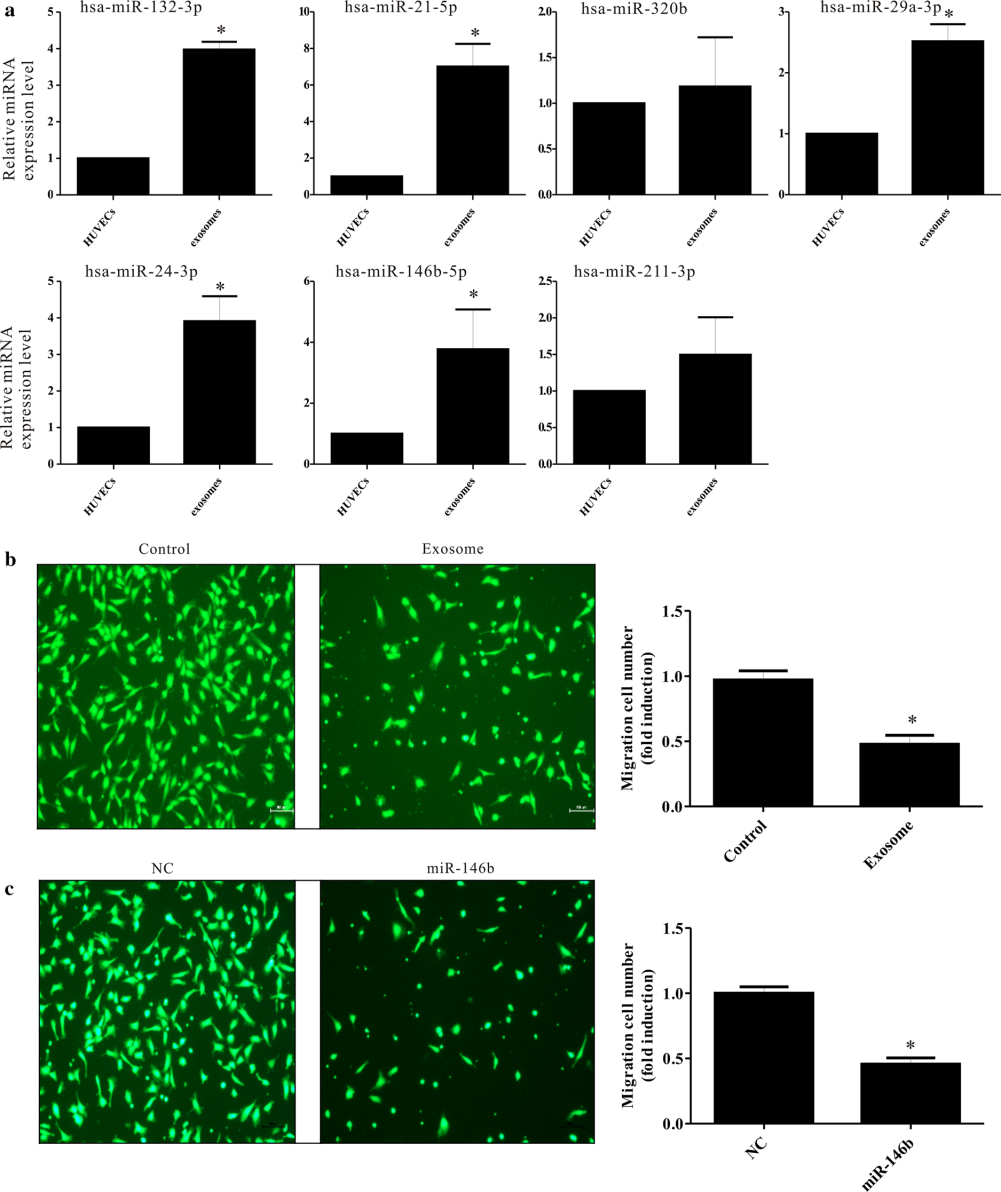
The expression of endothelial cell miRNA after co-culture with exosomes and miR-146b-5p can inhibit the migration of endothelial cells. **a** Quantification of individual miRNAs in HUVECs with or without exosomes derived from tumor-associated macrophages. The *y-axis* represents the relative miRNA expression level. The results are presented as the mean ± SD. miRNA expression in HUVECs with or without exosomes derived from tumor-associated macrophages. When HUVECs were co-cultured with exosomes derived from tumor-associated macrophages, a modest but statistically significant increase of miRNA expression was observed for miR-146b (*P < 0.05), miR-21 (*P < 0.05), miR-24 (*P < 0.05), and miR-132 (*P < 0.05). **b**, **c** Migration assay: the number of cells that migrate through the membrane to the lower chamber was measured with calcein-AM (*green*).Cells in the lower chamber were counted in three random microscopic fields using an inverted microscope. The ability of cells to migrate is significantly suppressed by the addition of exosomes derived from TAMs or by the mimics of hsa-miR-146b-5p

To demonstrate the uptake of exosome-derived miRNAs into recipient cells, we analyzed the expression of miRNAs in HUVECs with or without TAM-derived exosomes. We found significant increases of hsa-miR-132-3p, hsa-miR-21-5p, hsa-miR-320b, hsa-miR-29a-3p, hsa-miR-24-3p, hsa-miR-146b-5p, and hsa-miR-211-3p in HUVECs that were cultured with exosomes derived from TAMs compared with HUVECs without exosomes (Fig. 3a), suggesting that the extracellular miRNAs carried by TAM-derived exosomes can be transferred into HUVECs.

### TAM-derived exosomes suppress HUVEC cell migration

To investigate whether HUVECs were affected by exosomes, the migration of HUVECs was evaluated with 8-mm pore Transwell assays. Migrated cells (the ratio of cells that migrated to the bottom side) were reduced by approximately one half for HUVECs cultured with exosomes derived from TAMs (P < 0.05, Fig. 3b). These findings indicate that exosomes derived from TAMs suppress HUVEC migration.

miR-146b has been shown in a variety of tumors, including glioma, breast cancer, and thyroid cancer, to inhibit tumor metastasis or improve tumor radiosensitivity [10, 11]. Studies have also demonstrated that bone marrow stromal stem cells can transport miR-146b via exosomes to inhibit the proliferation of glioma cells [12]. To demonstrate the effect of miR-146b-5p on the migration of HUVECs, miR-146b-5p mimics or the miRNA negative control were transfected into HUVECS. Compared to the negative control, we found that the transfer of miR-146b-5p into HUVECs suppressed migration (P < 0.05, Fig. 3c). Collectively, the exogenous, TAM exosome-derived miR-146b-5p suppressed endothelial cell migration.

### Exosomal miR-146b-5p suppresses TRAF6 expression in HUVECs, and the inhibition of TRAF6 suppressed the migration in HUVECs

**Fig. 4:**
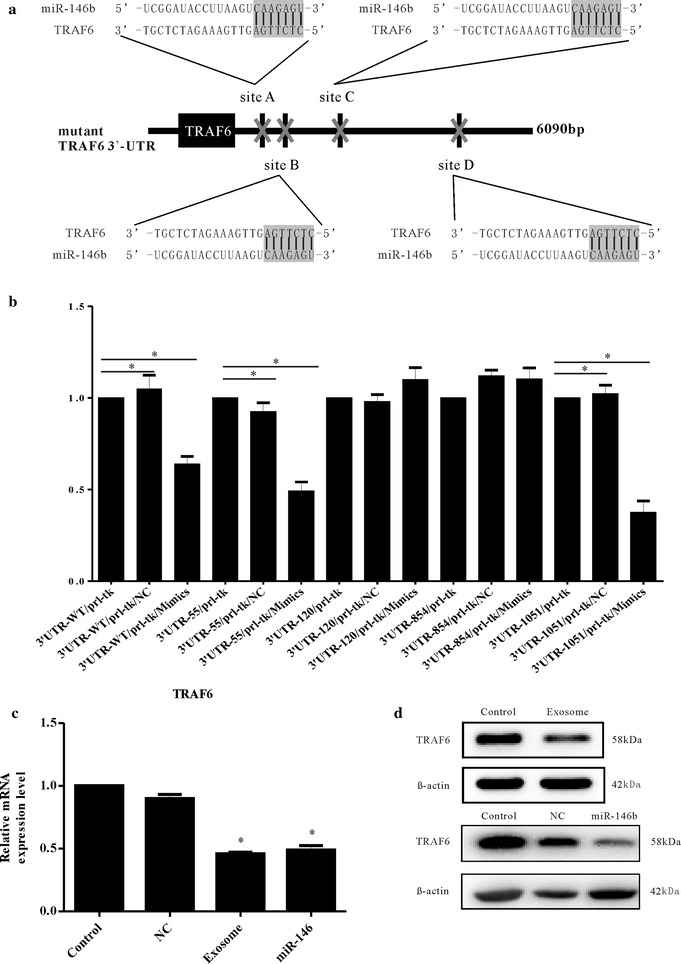
Exosomal miR-146b-5p targets the TRAF6 gene of endothelial cells. A luciferase reporter revealed that miR-146b-5p that could regulate TRAF6 expression. **a**, **b** Combinations of predicted miRNA recognition sites (MREs) for each putative target transcript of miR-146b-5p were cloned into the luciferase reporter vector and transfected into HUVECs along with the indicated miRNA mimics. The mean ± SD of three independent experiments is shown, and statistical significance is indicated by *(P < 0.05). **c**, **d** The expression of TRAF6, measured by qRT-PCR and Western blots, was significantly suppressed by the addition of exosomes derived from TAMs or the mimics of hsa-miR-146b-5p

Target scan software predicted TRAF6 as a target gene of miR-146b-5p. To test this prediction, a luciferase reporter assay was performed. miR-146b-5p decreased the luciferase activity of Luc-TRAF6-3′ UTR and had a minimal effect on the negative control (Fig. 4a, b). Next, we set up experimental groups in which HUVECs were co-cultured with exosomes derived from TAMs or miR-146b-5p mimics were transfected into HUVECs. In contrast, HUVECs were treated with PBS or miR-negative control mimics were transfected into HUVECs for control groups. The expression of TRAF6 was detected in experimental groups and control groups by real-time PCR and Western blots. The results showed that in experimental groups, the expression of TRAF6 was significantly suppressed, indicating that exosomal miR-146b-5p suppresses TRAF6 expression in HUVECs (Fig. 4c, d).

**Fig. 5:**
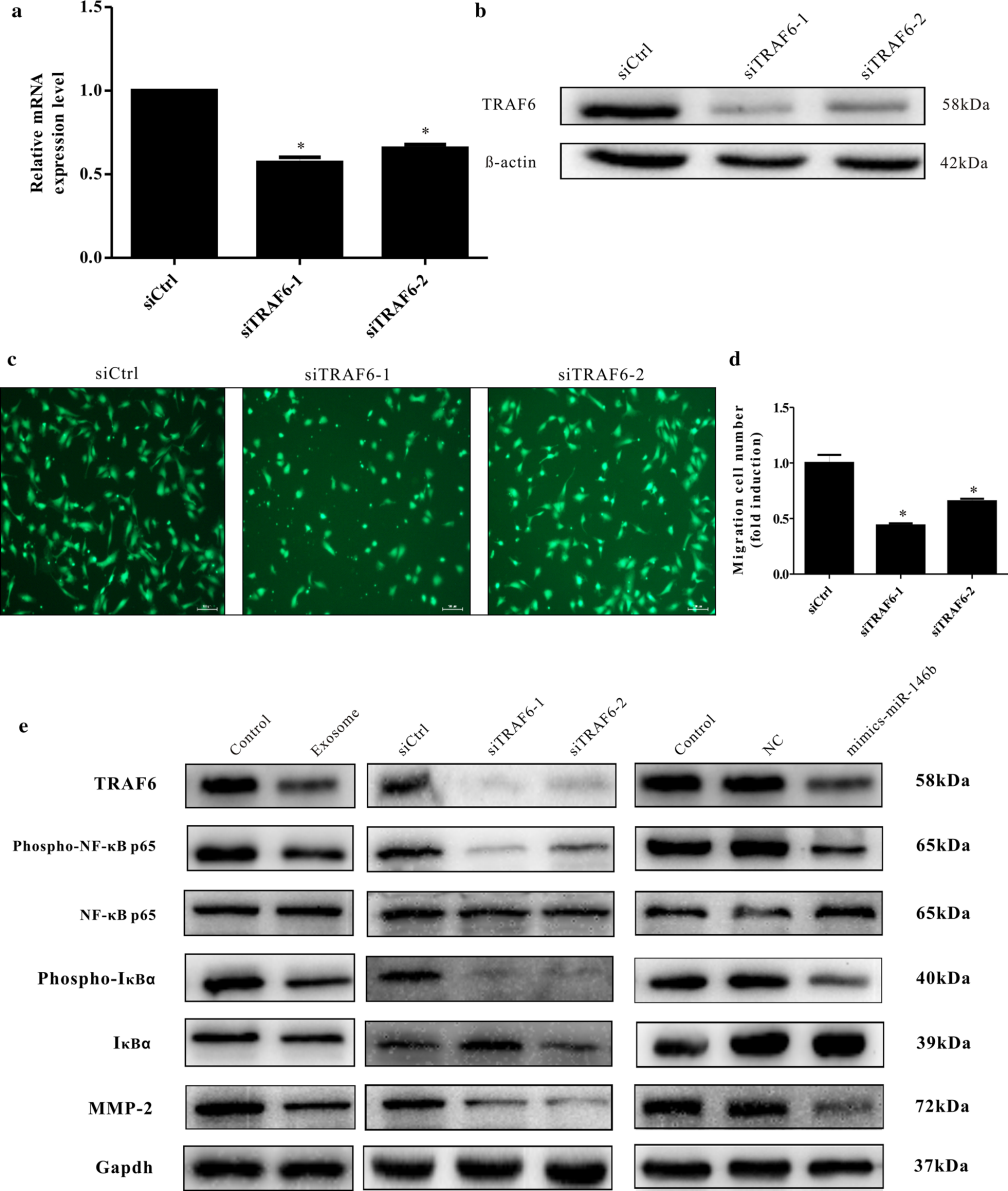
Regulation of TRAF6 expression and the related signaling pathway by TAM-derived exosomes. **a**, **b** TRAF6 gene silencing efficiency of EC cells by siTRAF6-1 and siTRAF6-2. **c**, **d** TRAF6 depletion by siTRAF6-1 and siTRAF6-2 reduced the migration of HUVECs. The mean ± SD of three independent experiments is shown, and statistical significance is indicated by *(P < 0.05). **e** A representative immunoblot of TRAF6, phosphorylated(p-)NF-κB, total NF-κB, phosphorylated(p-)IκBα, total IκBα, and MMP-2 in HUVECs treated with TAM-derived exosomes, miR-146b-5p mimics, or siTRAF6

Furthermore, when TRAF6 was depleted in HUVECs using siRNA (Fig. 5a, b), a significant reduction in migration (Fig. 5c, d) was found. The migration of HUVECS decreased by 50 percent in the presence of siTRAF6-1 and decreased approximately 35 percent in the presence of siTRAF6-2. Overall, these data show that TRAF6 plays a causal role in HUVEC migration.

### Exosomal miR-146b-5p suppresses the migration of HUVECs via TRAF6/NF-κB/MMP2

TRAF6 is a signal transducer in the NF-κB pathway. To explore whether TAM-derived exosomes could modulate the migration of HUVECs through the NF-κB pathway, NF-κB phosphorylation was assessed in HUVECs treated with exosomes (60 µg/ml). The activation of NF-κB phosphorylation was observed in HUVECs when they were incubated with TAM-derived exosomes (Fig. 5e). Matrix metalloproteinase 2 (MMP-2) is a member of a family of proteolytic enzymes that can enhance endothelial cell migration. We detected decreased expression of MMP-2 in HUVECs when they were incubated with TAM-derived exosomes (Fig. 5e). Western blotting revealed that the expression of TRAF6 was inhibited upon treatment with TAM-derived exosomes and that NF-κB phosphorylation was decreased in HUVECs; additionally, MMP-2 expression was decreased. NF-κB phosphorylation and MMP-2 expression were also measured after treatment with miR-146b-5p mimics or siTRAF6. We obtained the same results as with exosome incubation. These findings indicate that exosomal miR-146b-5p can suppress the migration of HUVECs via TRAF6/NF-κB/MMP2.

### EOC-derived exosomes transfer lncRNAs that restore the migration of endothelial cells suppressed by TAM-derived exosomes

**Fig. 6:**
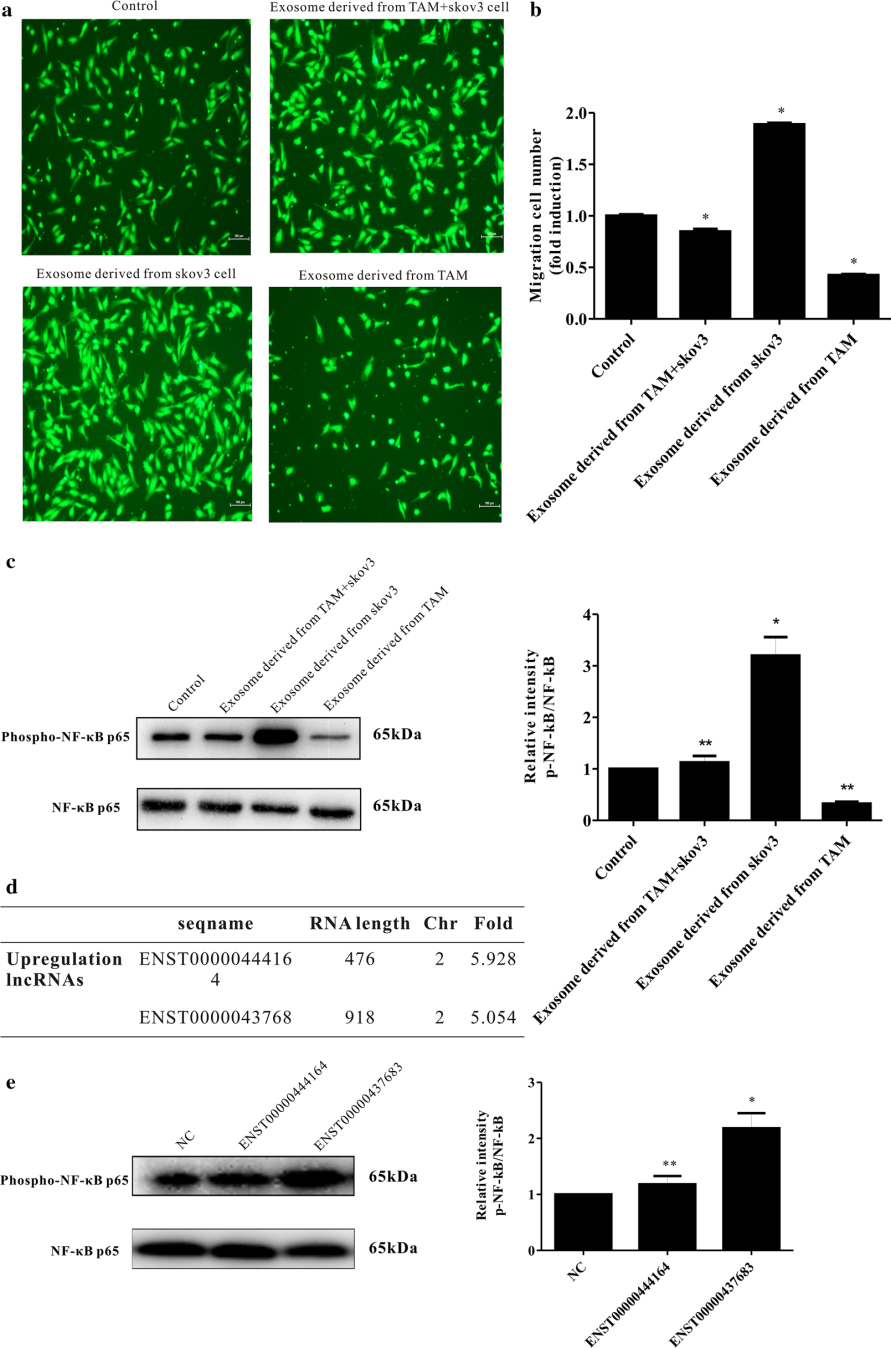
EOC-derived exosomes reverse the suppression of HUVEC migration by TAMs. **a**, **b** TAM-derived exosomes and EOCSKOV3 cell exosomes stimulate HUVECs. The inhibition of endothelial cell migration by TAM-derived exosomes was reversed, however, by the direct effect of SKOV3 exosomes in promoting endothelial cell migration. **c** NF-κB phosphorylation was inhibited after incubation with exosomes derived from SKOV3 cells. **d** 2 lncRNAs identified as potential NF-κB pathway-associated genes in EOCSKOV3 cell exosomes. **e** A representative immunoblot of phosphorylated(p-)NF-κB and total NF-κB in HUVECs overexpressing the two lncRNAs

Our previous work suggested that the co-culture of tumor-associated macrophage supernatant with EOCs could promote endothelial cell migration [3]. Here, we further showed that exosomes secreted from TAMs in the absence of EOC stimulation could suppress the migration of endothelial cells. To investigate whether macrophage function changes in the presence of exosomes derived from the EOC microenvironment, SKOV3-derived exosomes were added into this co-culture system. Interestingly, the inhibition of endothelial cell migration was reversed remarkably (Fig. 6a, b). Additionally, the phosphorylation of NF-κB was inhibited (Fig. 6c).

lncRNAs are associated with tumor cell proliferation, angiogenesis, and metastasis [13, 14]. We verified 2 lncRNAs as potential NF-κB pathway-associated genes in a previous study analyzing SKOV3-derived exosome arrays (Fig. 6d). These two lncRNAs were overexpressed and associated with NF-κB phosphorylation in HUVECs (Fig. 6e). Based on Fig. 6e, we deduced that lncRNAs may contribute to the restoration of the endothelial cell migration.

## Discussion

It is known that wide spread implantation and distant metastasis along the peritoneum of EOC are major causes of the poor long-term survival [15]. Tumor angiogenesis depends on the proliferation and migration of vascular endothelial cells after stimulation by various factors, thereby promoting tumor invasion and metastasis. Considerable studies show that the tumor microenvironment plays an important role in tumor progression and metastasis. Studies showed that when macrophages are exposed to a tumor microenvironment that overexpresses IL-4 and IL-10, the macrophages are polarized and differentiate into M2 macrophages, which are also known as tumor associated macrophages (TAMs). TAMs do not exert anti-tumor activities and are involved in tumor progression and angiogenesis [16]. Our previous work suggested that TAMs and endothelial cells interact with one another; under EOC stimulation, these TAMs could promote endothelial cell proliferation and migration to establish angiogenesis [3]. However, the exact mechanism of these three cell types’ interactions and the crosstalk among them remain unknown.

Most recently, exosomes have been identified as very important mechanisms for crosstalk between various cells. In tumor microenvironment, tumor cells and TAMs are the most important cell sources of exosomes. Our study suggested that TAM-derived exosomes can inhibit the migration of endothelial cells via the microRNA in it. MicroRNAs (miRNAs), small (21–25 nucleotides in length), non-protein-coding RNA transcripts, effectively regulate gene expression after gene transcription. Reports shows that mature miRNAs account for 41.7% of all RNA in exosomes [17]. In 2007, Valadi et al. [18]. first reported that mouse and human mast cell line-derived exosomes carried miRNA that could be transferred from one cell to another to impact important biological functions in the recipient cell. Since then, another study found that macrophage-derived exosomes can transfer miRNA into other recipient cells to mediate a regulatory role [19]. Gallo et al. [20] confirmed that exosomes are rich collectives of miRNA that can be used as reliable carriers for miRNA research. We assessed the effect of exosomes on the expression of endothelial cell miRNA after co-culture with exosomes and found the increased expression of 7 miRNAs (Fig. 3a).

miR-146b has been shown in a variety of tumors including glioma, breast cancer, and thyroid cancer to inhibit tumor metastasis or improve tumor radiosensitivity [10, 11], and miRNA mimic transfection showed that miR-146b-5p could inhibit the migration of endothelial cells (Fig. 3c). Through complementary base pairing to a specific target in the 3′ UTR of mRNA, microRNAs degrade mRNA or inhibit the translation of mRNA to reduce the expression level of target genes, and thus playing a role in regulating cell growth, differentiation, proliferation, apoptosis, and other physiological processes [21]. miR-146b-5p targeted the TRAF6 gene of endothelial cells, and the decreased expression of TRAF6 influenced endothelial cell migration (Fig. 5a–d) through Matrix metalloproteinase-2 (MMP2). MMP2 is a member of the proteolytic enzyme family. Multiple cells, including fibroblasts, macrophages, endothelial cells, and malignant cells, secrete inactive MMP2 that can be activated by specific activators. MMP2 can degrade the extracellular matrix, which increases the spaces between cells and provides a favorable environment for the formation of tumor blood vessels, and it can also enhance endothelial cell migration. We found that TAM-derived exosomes targeted the miR-146b-5p/TRAF6/NF-kB/MMP2 pathway to suppress endothelial cell migration (Fig. 5e).

Tumor necrosis factor receptor-associated factor 6 (TRAF6) is a signal transducer in the nuclear factor-κB (NF-κB) pathway. Upon the stimulation of cells by various agonists, such as tumor necrosis factor α (TNFα) and interleukin 1β (IL-1β), IκB proteins are rapidly phosphorylated by an IκB kinase (IKK) complex and then degraded by the ubiquitin (Ub)-proteasome pathway. Following IκB degradation, NF-κB translocates into the nucleus where it regulates the expression of a wide spectrum of genes involved in immunity, inflammation, apoptosis, and other cellular processes [22]. Some researchers have found that TRAF6 is closely related to tumor development. As a very important ubiquitin E3 ligase, TRAF6 may induce the ubiquitination of the AKT oncogene. In a nude mouse tumorigenicity model, stable TRAF6 knockdown cells had lower tumorigenic potential than control cells, which suggested that TRAF6 is an oncogene [23]. Meanwhile, the inhibition of TRAF6 expression can significantly inhibit the proliferation and invasion of pancreatic cancer, breast cancer, lung cancer, esophageal cancer, multiple myeloma, and other cells [24, 25].

Some studies have found that TAM culture supernatant can promote endothelial cell migration, but our study found that exosomes isolated from TAMs in the ascites of epithelial ovarian cancer (EOC) inhibit the migration of endothelial cells. This finding shows that macrophage function can be changed in the presence of tumor cells. Ovarian cancer cell-derived exosomes participate in the regulation of TAMs and endothelial cells, as revealed through migration assays. TAM-derived exosomes and SKOV3 cell exosomes stimulate HUVEC cells. The inhibition of endothelial cell migration by TAM-derived exosomes was reversed, however, by the direct effect of SKOV3 exosomes in promoting endothelial cell migration (Fig. 6b). We speculate that certain substances in EOC-derived exosomes reverse the suppression of HUVEC migration by TAMs in the tumor microenvironment. Long noncoding RNAs (lncRNAs) are RNA transcripts that are more than 200 nt long but have little protein-coding potential. Within the last few years, thousands of lncRNAs have been implicated in biological processes. For example, lncRNA-p21 has been shown to act in trans or in cis to regulate target gene expression [26]. In our study, we found 2 lncRNA as potential NF-κB pathway-associated genes (Fig. 6d). This observation revealed the important role of exosomes from EOC cells in tumor micro-environment. It gets a glimpse of the complicated network of stromal cells in tumor-microenvironment. We may hypothesis that Before the tumor cells seeded, the TAM cell suppressed the angiogenesis, released some immune factor to cause chronic inflammation according other studies. When tumor cells come, endothelial cells show more active, and supplies more nutrients for tumor cells. Since lacking of advanced technology to confirm the direct contact between these LncRNA and NF-κB pathway-associated genes, we did not figure out how these two lncRNA regulate the NF-κB pathway. However, with the development of the Gene technique, we will study the mechanism how these two lncRNAs control the phosphorylation of NF-κB in vivo, and find a biological tool to interdict the NF-κB pathway only in the endothelial cells in tumor environment.

## Conclusion

We demonstrated that the lncRNA carried by exosomes derived from SKOV3 cells could activate the phosphorylation of NF-κB in HUVECs. Perhaps this finding helps explain why EOC-derived exosomes reverse the suppression of HUVEC migration by TAMs.

## Methods

### Patients

Between October and December 2014, ascitic fluid was obtained from 5 patients with EOC undergoing debulking surgery at Shanghai First Maternity and Infant Hospital, Tongji University. Serum was prepared from blood donated by 5 volunteers and 5 patients with EOC. The study protocol was approved by the Institutional Review Board of Shanghai First Maternity and Infant Hospital, Tongji University according to the Ethics Committee of Shanghai First Maternity and Infant Hospital. Informed consent was obtained from patients or their guardians.

### Patient samples

Blood samples from 5 healthy controls and 5 pre-therapy EOC patients were collected in a Vacutainer blood collection tube (BD, USA). The tubes were centrifuged at 1500*g* for 10 min; then, the supernatants were delivered to new tubes and stored at −80 °C until processing. Specimens were obtained from the Shanghai First Maternity and Infant Hospital, Tongji University (Shanghai, China) according to the Ethics Committee of Shanghai First Maternity and Infant Hospital. Informed consent was obtained from patients or their guardians.

### The separation and identification of tumor-associated macrophages

TAMs were separated from the ascites of epithelial ovarian cancer via CD14 magnetic beads and then cultured in RPMI 1640 Gibco supplemented with 10% FBS, 100 U⁄ml penicillin, and 100 U⁄ml streptomycin at 37 °C in 5% CO_2_.

TAMs were isolated, and the percentages of CD206+ and HLA-DR+ cells were analyzed by FACS. TAMs exhibited higher expression of CD206 and lower expression of HLA-DR.

### Exosome isolation

To isolate exosomes derived from TAMs associated with epithelial ovarian cancer and human EOCSKOV3 cells, which were obtained from FuHeng BIO (Shanghai, China), cells were first cultured in RPMI-1640 for 48 h. We centrifuged the cell supernatants twice (2000*g* for 10 min, then 2500*g* for 30 min to deplete the cells or fragments), added the total exosome isolation kit (Life technology) overnight, and then centrifuged at 10,000*g* for 1 h. Exosomes were resuspended in PBS (Gibco) and stored at −80 °C. The exosome concentration was detected by the BCA Protein Assay.

### Flow cytometry analysis

TAMs were isolated and used to analyze the cytomembrane expression of CD206 and HLA-DR by flow cytometry. The data are expressed as the percentages of immunocytes with positive markers.

### Immunofluorescence microscopy for the detection of HUVEC ingestion of exosomes derived from tumor-associated macrophages

Exosomes were labeled with PKH67(Sigma-Aldrich, St. Louis, MO, USA), a green fluorescent dye with long aliphatic tails that localizes in lipid regions of the exosome membranes, for 5 min at 37 °C. Labeled exosomes were washed 3 times with PBS and centrifuged at 80,000*g* for 2 h. Cells were cultured for 24 h, and HUVECs were collected for microscopy to detect exosomes secreted by TAMs.

### Transmission electron microscopy

Exosome pellets were dissolved in PBS buffer, dropped on a carbon-coated copper grid, and then stained with 2% uranyl acetate. The samples were observed using a J Tecnai G2 F20 ST transmission electron microscope.

### Co-culture system of Exosomes and HUVECs

TAMs that were separated from the ascites of epithelial ovarian cancer and human EOC-SKOV3 cells were cultured in RPMI-1640 for 48 h. Human umbilical vein endothelial cells (HUVECs) were isolated in the Central Laboratory of Shanghai First Maternity and Infant Hospital. Exosomes were isolated as above. Exosomes were combined with HUVECs at 60 ng/ml of culture medium for 72 h.

### Transfection of mimics and siRNA

Mimics and the siRNA targeting TRAF6 (si-TRAF6)were purchased from Shanghai Jima Company. Lipo2000 (Life Technology) was used to transfer mimics into HUVECs according to the manufacturer’s instructions. After 48 h, the transfection efficiency of mimics and siRNA was detected. The siRNA sequences used were as follows:

siTRAF6-1, forward: 5′-GGGUACAAUACGCCUUACATT-3′

reverse: 5′-UGUAAGGCGUAUUGUACCCTT-3′

siTRAF6-2, forward: 5′-GCAGUGCAAUGGAAUUUAUTT-3′

reverse: 5′-AUAAAUUCCAUUGCACUGCTT-3′

### HUVEC migration assay

Transwell chambers (6.5 mm) (Corning Costar, Cambridge, MA, USA) with 8.0-µm pore polycarbonate membranes were coated with Matrigel. HUVECs (20,000 cells/well) were incubated in the upper chamber at 37 °C in 5% CO_2_ and allowed to migrate for 8 h toward the lower chamber. Some HUVECs were co-cultured with exosomes for 72 h. The number of cells that migrated through the membrane to the lower chamber was measured after 8 h with calcein-AM (Invitrogen, C3100MP; 50 µg).Cells in the lower chamber were counted in three random microscopic fields using an inverted microscope (Nikon, Japan).

### 3′ UTR luciferase assay

The 3′ untranslated region (3′ UTR) reporter plasmid for the TRAF6 gene was generated by cloning the 3′ UTR downstream of the luciferase open reading frame (Hanyin Biotechnology, Shanghai, China), Then, the 3′ UTR luciferase reporter plasmid together was transfected with the miR-146b-5p mimics or the miR-negative control using Lipofectamine 3000 (Invitrogen, CA, USA) into HUVECs in a 24-well plate (500 ng luciferase plasmid plus 50 nMmiR-146b-5p mimics). A constitutively expressed Renilla luciferase was co-transfected as a normalizing control. After 24 h of incubation, Firefly and Renilla luciferase activities were sequentially measured using the Dual-Glo Luciferase Assay system (Promega, Madison, WI, USA).

### RNA extraction and MicroRNA profiling by RT-PCR

RNA from exosomes was isolated and enriched with a Total Exosome RNA and Protein Isolation Kit (Invitrogen, CA, USA) according to the user’s guide, and the total RNA of HUVECs stimulated with the exosomes (60 µg/ml) after 48 h was extracted using TRIzol (Invitrogen, CA, USA). The miScript Reverse Transcription Kit and miScript SYBR Green PCR Kit (Qiagen GmbH, Hilden, Germany) were used to reverse transcribe and quantitatively detect miRNAs according to the manufacturer’s protocol. Human RNU6B was used to normalize miRNA expression. The data were calculated using the 2-ΔΔCT method.

The primer oligonucleotide sequences were as follows:

hsa-miR-132-3p: 5′-TAACAGTCTACAGCCATGGTCG-3′

hsa-miR-21-5p: 5′-TAGCTTATCAGACTGATGTTGA-3′

hsa-miR-320d: 5′-AAAAGCTGGGTTGAGAGGA-3′

hsa-miR-29a-3p: 5′-TAGCACCATCTGAAATCGGTTA-3′

hsa-miR-24-3p: 5′-TGGCTCAGTTCAGCAGGAACAG-3′

has-miR-146b-5p: 5′-TGAGAACTGAATTCCATAGGCT-3′

hsa-miR-211-5p: 5′-TTCCCTTTGTCATCCTTCGCCT-3′

U6: 5′-CAAGGATGACACGCAAATTCG-3′

### Quantitative RT-PCR

Total RNA was isolated from HUVECs incubated with exosomes (60 µg/ml),miR-146b-5p mimics, or siRNA targeting TRAF6 (n = 3) for 48 or 72 h, and cDNA was transcribed using a PrimeScript™ RT Reagent Kit (Perfect Real Time) (Takara, Japan). Real-time quantitative RT-PCR was performed using the StepOnePlus™ Real-Time PCR System (Invitrogen, CA, USA) to detect TRAF6 mRNA. Amplification was performed with SYBR^®^ Premix Ex Taq™ (TliRNaseH Plus) (Takara, Japan), and the 2-ΔΔCT method was used to calculate gene expression with β-actin as an internal reference. The primer sequences used were as follows:

TRAF6 primers, forward: 5′-ATGCGGCCATAGGTTCTGC-3′,

reverse: 5′-TCCTCAAGATGTCTCAGTTCCAT-3′;

β-actin primers, forward: 5′-CCTGGCACCCAGCACAAT-3′,

reverse: 5′-GGGCCGGACTCGTCATACT-3′.

### Western blotting analysis

Total protein was isolated from HUVECs treated with exosomes (60 µg/ml), miR-146b-5p mimics, or siRNA targeting TRAF6 (n = 3) for 48 or 72 h. The membranes were blocked with 5% bovine serum albumin for 2 h and incubated with antibodies against TRAF6 (1:2000; Abcam, USA), NF-κB Pathway Sampler Kit(1:1000, CST, USA), and GAPDH (1:5000; Abcam, USA) at 4 °C overnight. Peroxidase-linked secondary anti-rabbit (1:2000; CST) or anti-mouse antibodies (1:2000, CST) were used to detect the bound primary antibodies, and the blotted proteins were visualized using an enhanced chemiluminescence kit (Pierce Biotechnology). The intensity of protein bands was quantified using the Image J software (National Institutes of Health, MD, USA). The relative expression of target proteins was described as a ratio relative to the expression of GAPDH, and statistical data from at least three experiments were graphed.

### Statistical analysis

Statistical analyses were performed with SPSS19.0. The data are expressed as the mean ± SD. The Mann–Whitney test, one-way ANOVA, Fisher’s exact test, and Student’s *t* test were used to determine P values. Continuous variables in figures are shown as the mean ± SEM. A P < 0.05 was considered statistically significant.

## Additional file

**Additional file 1.** The animation of the mechanism that how EOC reversed the suppression of endothelial cell migration by TAM-derived exosomes.
